# Hair Calcium Levels in Relation to Coronary Artery Disease Severity and Systemic Inflammation Markers: A Pilot Study

**DOI:** 10.3390/jcm14134537

**Published:** 2025-06-26

**Authors:** Ewelina A. Dziedzic, Aleksandra Czernicka, Jakub S. Gąsior, Anna Szamreta-Siwicka, Beata Wodejko-Kucharska, Paweł Maciński, Anna Arbaszewska, Konrad Adler, Andrzej Osiecki, Wacław Kochman

**Affiliations:** 1 Cardiovascular Clinic, Centre of Postgraduate Medical Education, 01-813 Warsaw, Poland; 2 Department of Pediatric Cardiology and General Pediatrics, Medical University of Warsaw, 02-091 Warsaw, Poland; 3 Department of Cardiology, Bielanski Hospital, 01-809 Warsaw, Poland; 4 Faculty of Medicine, Collegium Medicum, Cardinal Stefan Wyszynski University of Warsaw, 01-938 Warsaw, Poland

**Keywords:** coronary artery disease, calcium, inflammation, coronary atherosclerosis

## Abstract

**Background**: Coronary artery disease (CAD) is a leading global cause of mortality. The role of calcium (Ca), a key metabolic and structural element, in atherosclerosis and inflammation remains unclear. Ca influences immune cell function and is a component of atherosclerotic plaques. Hair analysis reflects long-term mineral exposure and may serve as a non-invasive biomarker. **Objectives**: This pilot study aimed to investigate the association between hair Ca levels and acute coronary syndrome (ACS), and to evaluate correlations with the Systemic Inflammatory Index (SII), Systemic Inflammatory Response Index (SIRI), and selected CAD risk factors. **Methods**: Ca levels were measured in hair samples from patients undergoing coronary angiography for suspected myocardial infarction. Associations with ACS diagnosis, Syntax score, SII, SIRI, and CVD risk factors were analyzed. **Results**: Serum calcium levels were not significantly associated with the presence of acute coronary syndrome (ACS) (*p* = 0.392) or with its clinical subtypes, including ST-elevation myocardial infarction (STEMI), non-ST-elevation myocardial infarction (NSTEMI), and unstable angina (UA) (*p* = 0.225). Diagnosis of ACS was linked to higher SII (*p* = 0.028) but not SIRI (*p* = 0.779). Ca levels correlated negatively with Syntax score (R = −0.19, *p* = 0.035) and SII (R = −0.22, *p* = 0.021) and positively with HDL-C (R = 0.18, *p* = 0.046). **Conclusions**: Hair calcium content may reflect subclinical inflammation and CAD severity. Although no direct link to ACS was observed, the associations with SII, HDL-C, and Syntax score suggest a potential diagnostic role which should be further explored in larger, well-controlled studies.

## 1. Introduction

The dynamic progression of coronary artery disease forms the basis for its classification into two primary categories: chronic coronary syndrome (CCS) and acute coronary syndrome (ACS). CCS is characterized by persistent inflammation within the walls of the coronary arteries [1]. This condition results in endothelial dysfunction, activation of vascular smooth muscle cells and platelets, lipid accumulation, and alterations in extracellular matrix metabolism. These processes collectively promote the formation of atherosclerotic plaques [2]. Progressive changes in these plaques may lead to rupture or erosion, events that can precipitate ACS [3].

Calcium is an essential mineral in the human body. Research indicates that serum calcium levels undergo significant fluctuations, often related to changes in albumin concentration or hydration status [4]. In contrast, hair calcium concentration serves as a more reliable marker of long-term exposure, primarily due to its lower susceptibility to confounding factors and its better reflection of the total body burden. This is because trace elements accumulate in hair follicles at concentrations approximately ten times higher than those found in urine, blood, or plasma [5]. Moreover, hair sample collection is simple, painless, and non-invasive, making it an ideal method for such research [6]. Calcium plays critical roles in neural transmission, blood coagulation, enzymatic modulation, and bone mineralization [7]. It also regulates cardiac muscle contraction; however, its full impact on the cardiovascular system remains to be fully elucidated [8]. The role of calcium in the regulation of endothelial cell function, which is crucial in the pathogenesis of atherosclerosis, is well documented [9]. The endothelial glycocalyx, a specialized surface layer essential for maintaining vascular barrier integrity, is regulated by various ion channels and receptors, including transient receptor potential (TRP) channels, which control intracellular Ca^2+^ levels [10]. Elevated serum calcium can activate the calcium-sensing receptor (CaSR) on endothelial cells, leading to an increase in intracellular Ca^2+^ concentrations and subsequent stimulation of the NLRP3 inflammasome [11]. This process results in the release of proinflammatory cytokines such as IL-1β and IL-18, thereby contributing to vascular inflammation and dysfunction [12]. Calcium also plays a role in regulating vascular tone through endothelium-derived vasoactive mediators such as nitric oxide (NO) and prostacyclins [13]. Additionally, elevated calcium levels can enhance the synthesis of endothelin-1 (ET-1), a key mediator in the development of atherosclerosis and acute coronary syndromes (ACSs) [14]. Moreover, calcium signaling may contribute to endothelial repair by facilitating the recruitment of neighboring endothelial cells, as well as circulating endothelial progenitor cells [15]. Importantly, hair calcium levels may indirectly reflect cumulative calcium exposure relevant to endothelial cell activity, especially considering that endothelial cells are capable of undergoing endothelial-to-mesenchymal transition (EndMT) and acquiring osteogenic properties in response to prolonged calcium dysregulation [16]. This phenotypic shift contributes not only to vascular calcification but also to sustained endothelial dysfunction and inflammation. In contrast to serum levels, which are subject to short-term fluctuations, hair calcium reflects intracellular accumulation of this element over time [17], thereby offering a unique, non-invasive insight into processes potentially involved in endothelial activation and damage.

Coronary artery calcification plays a key role in the development of CAD, serving not only as an indicator of progressive atherosclerosis but also, through its influence on the function of immune system cells, as a significant determinant of plaque stability [18,19,20]. In neutrophils, calcium plays a crucial role in degranulation and cell adhesion [21]. It also participates in producing reactive oxygen species (ROS) during the oxygen burst, and it aids in the formation of neutrophil extracellular traps (NETs) and the secretion of various cytokines [22]. In lymphocytes, it is crucial for their activation, proliferation, differentiation, survival, apoptosis, and production of cytokines and chemokines such as IL-10 [23,24,25]. Calcium channels play a key role in lymphocyte development, especially regarding negative selection, homeostasis maintenance, and the polarization of helper lymphocytes [23]. The primary calcium channels in lymphocytes are Ca^2+^ release-activated Ca^2+^ (CRAC) channels, which regulate their motility and are essential for the degranulation of cytotoxic CD8+ lymphocytes [24]. Calcium ions are essential in the process of cell lysis induced by cytotoxic T lymphocytes and NK cells [26]. It has also been shown that during inflammation, the calcium gradient regulates monocyte chemotaxis via the calcium-sensing receptor (CaSR). This element activates the NLRP3 inflammasome in monocytes/macrophages via the CaSR receptor, enhancing lysosomal activity in cells related to the inflammasome in the presence of lipopolysaccharide [27]. Calcium ions in lysosomes play a key role in signal transduction, maintaining organelle homeostasis, and promoting lysosome fusion. Calcium is a factor that releases various particles and proteins from macrophages in inflamed tissues, which originate from circulating monocytes [28]. Importantly, the S100A8 and S100A9 proteins stimulate the production of metalloproteinases MMP8 and MMP9, which play a crucial role in atherogenesis [29]. Calcium channels are key components in platelets, and their activation enhances various signaling mechanisms [30]. Their role is to produce secondary messengers, including platelet-derived thromboxane A2 (TxA2) and activating integrins [31]. Furthermore, calcium stimulates the production of reactive oxygen species (ROS), which also leads to the mutual activation of platelets [32].

Due to the pivotal role of inflammation in the pathogenesis of CAD, increasing attention has been directed toward easily accessible inflammatory biomarkers derived from complete blood counts, such as the Systemic Immune-Inflammation Index (SII) and the Systemic Inflammation Response Index (SIRI) [33]. SII, based on neutrophils, lymphocytes, and platelets, reflects the interplay between systemic inflammation and immune response activity [34,35]. Elevated SII values have been associated with the presence and severity of CAD [36,37,38], as well as with the incidence of major adverse cardiovascular events (MACEs) in patients following coronary interventions [39,40], with heart failure [41], and after cardiac surgery [42,43,44,45]. Similarly, SIRI—calculated using absolute monocyte, neutrophil, and lymphocyte counts [46]—has shown predictive value for cardiovascular disease (CVD) occurrence [47] and MACE risk in patients undergoing percutaneous coronary intervention (PCI) due to acute coronary syndrome (ACS) [48,49].

Importantly, while both indices have been independently validated in various inflammatory and cardiovascular contexts, they reflect different yet complementary immunologic mechanisms. SII captures innate immune activation and thrombopoietic involvement [(platelet × neutrophil)/lymphocyte] [50], while SIRI highlights monocyte-driven low-grade inflammation and myeloid lineage activation [(neutrophil × monocyte)/lymphocyte] [35,50].

Their combined analysis may therefore offer a more comprehensive evaluation of systemic immune status, capturing broader inflammatory pathways—including neutrophil–lymphocyte imbalance, chronic monocyte activity, and platelet-associated thrombo-inflammatory processes—which are implicated in early, subclinical stages of atherosclerosis and cardiovascular dysfunction [48,51]. Moreover, accumulating evidence suggests that SII and SIRI may respond differently across clinical phenotypes and metabolic states, underlining their potential complementary use for improved sensitivity, reduced diagnostic uncertainty, and enhanced cardiovascular risk stratification in both preventive and prognostic settings [48,52,53].

Based on evidence supporting the involvement of calcium in the pathogenesis of coronary artery disease, the present study aimed to explore the relationship between calcium levels in hair samples and the diagnosis of chronic and acute coronary syndromes in patients with normal serum calcium concentrations. Additionally, we evaluated the association between hair calcium concentration and inflammatory markers such as SII and SIRI, as well as established cardiovascular risk factors, including age, sex, smoking, hypertension, diabetes, dyslipidemia, and body mass index. Importantly, we do not interpret hair calcium as a functional equivalent of intracellular calcium signaling. Rather, we consider it a non-invasive, cumulative marker reflecting long-term systemic calcium handling, which may be potentially relevant to vascular dysfunction and the development of atherosclerosis.

## 2. Materials and Methods

**Figure 1:**
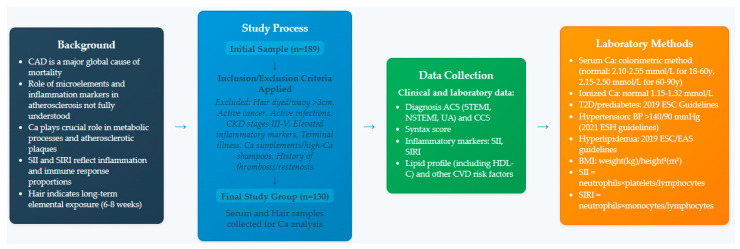
Flowchart of the study.

Figure 1 presents a flowchart of the study.

### 2.1. Study Population

The study included a total of 130 consecutive patients (36 women and 94 men) who met the inclusion criteria and were referred for coronary angiography due to suspected ACS. Patients diagnosed with CCS automatically served as the control group. Data for the study were collected from 2013 to 2017 at the Cardiology Department of Bielański Hospital in Warsaw, Poland. All participants were residents of Warsaw and reported no occupational exposure to calcium. The study received approval from the Bioethics Committee of the Medical University of Warsaw (approval number KB/124/2014) and was conducted following the principles of the Declaration of Helsinki. Each participant provided written consent to take part in the study.

### 2.2. Exclusion Criteria

Patients who dyed their hair or had wavy hair longer than 3 cm were excluded from the study. Other exclusion criteria included active cancer; active viral or bacterial infections; chronic kidney disease in stages III, IV, and V; elevated inflammatory markers; terminally ill patients; and those who take calcium supplements or use shampoos with high calcium content. Patients with a history of thrombosis or vascular restenosis were also excluded from the study.

### 2.3. ACS Diagnosis and Angiography

Patients in the study underwent coronary angiography, using either a radial or femoral approach, to evaluate the degree of coronary artery stenosis. Based on the results, patients were qualified for revascularization if necessary. The preferred method for this procedure was percutaneous coronary intervention (PCI) [54]. The diagnosis of ACS was made according to the European Society of Cardiology guidelines, which specify the diagnosis criteria as an increase in the concentration of a myocardial damage marker, specifically troponin, with at least one value exceeding the 99th percentile of the upper reference limit in combination with one of the following: symptoms of myocardial ischemia, new ischemic changes on the electrocardiogram (ECG), presence of a pathological Q wave on the ECG, confirmation of loss of cardiac viability through imaging studies, new regional myocardial contractility disorders related to ischemia, visualization of a thrombus in a coronary artery during angiography [55]. The severity of coronary atherosclerosis was assessed using the SYNTAX scale. This scale uses an algorithm that considers various factors, including the spatial distribution of lesions in the coronary arteries, the number of lesions, and their effect on hemodynamics. This assessment is crucial because the score derived from the SYNTAX scale serves as an independent predictor of adverse cardiovascular events during long-term follow-up. Furthermore, the results from the SYNTAX scale guide the choice of revascularization methods, determine the extent of the procedure, and inform postoperative care strategies [56,57].

### 2.4. Laboratory Tests and Clinical Assessment

Venous blood samples were analyzed within two hours of obtaining the material. Total calcium and ionized calcium levels in serum were calculated using a colorimetric method. All patients had both of these calcium parameters within the normal range using the standards for the local hospital laboratory, with the standards for total calcium being 2.10–2.55 mmol/L in patients aged 18–60 years and 2.15–2.50 mmol/L in the age group 60–90 years. The standards for ionized calcium were 1.15–1.32 mmol/L. The diagnosis of type 2 diabetes or prediabetes was made according to the 2019 ESC Guidelines on diabetes, prediabetes, and cardiovascular disease. The criteria for diagnosis include an increase in fasting blood glucose levels above 126 mg/dL (or ≥7.0 mmol/L) confirmed by two separate measurements, the presence of diabetes symptoms alongside a random blood glucose level of ≥200 mg/dL (or ≥11.1 mmol/L), or a blood glucose result of ≥200 mg/dL (or ≥11.1 mmol/L) after 120 min during an oral glucose tolerance test (OGTT) [58]. According to the 2019 ESC/EAS guidelines for treating dyslipidemia, hyperlipidemia was diagnosed in patients whose lipid profiles did not meet the therapeutic targets appropriate for their risk level [59]. Hypertension was defined as a blood pressure reading exceeding 140/90 mmHg, following the 2021 European Society of Hypertension practice guidelines [60]. Body mass index (BMI) was measured to evaluate the patient’s nutritional status based on their height and weight. BMI was calculated by dividing body weight in kilograms by the square of height in meters (kg/m^2^). A BMI value of 30 kg/m^2^ or higher was defined as obesity [61]. The value of subclinical inflammatory markers was calculated using the following formulas: SII = (neutrophil count) × (platelet count)/(lymphocyte count) and SIRI = neutrophil count) × (monocyte count)/(lymphocyte count) [62]. The necessary counts of individual immune system cells for these calculations were obtained by analyzing a complete blood count using an automatic blood counter.

### 2.5. Analysis of Hair Samples

The study used samples of undyed hair weighing approximately 0.2–0.3 g, collected from various locations on the back of the scalp, close to the skin. Samples were washed using non-ionic detergent (Triton X-100, Sigma Aldrich Sp. z.o.o., Poznań, Poland) water solution (1:100) in an ultrasonic bath for 5 min. Samples were rinsed with high-purity water, acetone, and water and then dried to constant mass. The dry samples of hair, 0.15 g each, were dissolved in 4 mL of 65% nitric acid (Merck, Darmastadt, Germany) and 1 mL of 30% hydrogen peroxide (Merck) in a closed polypropylene tube (8 mL). The next stage was incubation at 80 °C for 30 min in a microwave station. After cooling to room temperature, the samples were diluted to a final volume of 10 mL with Milli-Q water and then analyzed using an inductively coupled plasma optical emission spectrometry (ICP-OES) spectrometer (iCAP7400, Thermo Scientific, Waltham, MA, USA). To determine the final concentration of calcium in the solution, certified standards CGZN1 and CGCU1 (Inorganic Ventures, Christiansburg, VA, USA) were used for this element.

### 2.6. Statistical Analysis

Shapiro–Wilk tests were performed to assess the normality of the data, and Pearson’s chi-square test or Fisher’s exact test was used to determine differences between groups, depending on the distribution of the data. A Mann–Whitney test compared continuous variables between the two groups. Differences between groups were assessed using a nonparametric Kruskal–Wallis test with Dunn’s multiple comparisons test. The relationship between the selected variables was analyzed with a Spearman correlation coefficient (R). A *p*-value < 0.05 was considered statistically significant. Statistical significance was achieved when the two-sided *p*-value was less than 0.05. The software used for the analysis included Statistica 13 (StatSoft Inc., Tulsa, OK, USA), and Julius AI (https://julius.ai/) was utilized to draw the figures.

## 3. Results

### 3.1. Patient Characteristics

Results of 130 participants with suspected ACS were presented in the study. Table 1 (second column) presents characteristics for the whole group of patients. In addition to data presented in Table 1, the following percentage characteristics were observed: 29.2% of participants had a normal body weight, 40.0% were overweight, and 30.8% of patients were classified as obese. Active smoking during the study was declared by 29.2% of patients; 13.1% of patients had smoked in the past, and 57.7% of patients were non-smokers. Hypertension was present in 86.2% of patients. A history of type 2 diabetes mellitus (T2DM) or diagnosis during the current hospitalization was found in 31.5% of patients, pre-diabetes was found in 5.4% of patients, and no T2DM diagnosis was found in 63.1% of patients. Based on the lipid profile, hyperlipidemia was assessed in 92% of patients and diagnosed in 40.8%. A history of myocardial infarction (MI) was noted in 29.2% of patients. Comparison analysis between patients with stable CAD and ACS was also presented in Table 1. Table 2 presents comparison between patients with stable CAD and patients with STEMI (N = 31, 23.9%), NSTEMI (N = 19, 14.6%), and UA (N = 15, 11.5%).

### 3.2. Association Between Ca Level and Severity of CAD

**Figure 2:**
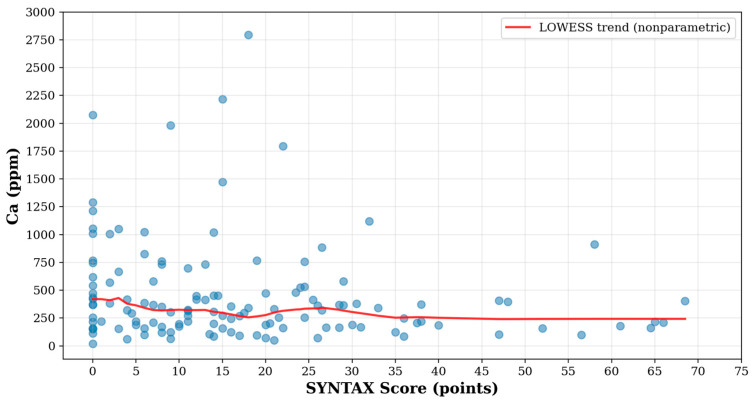
Correlation between Ca and the SYNTAX score.

There was a significant (negative) correlation between Ca and the SYNTAX score (R = −0.19, *p* = 0.035, Figure 2). The LOWESS (locally weighted scatterplot smoothing) trend line (appropriate for non-parametric data analysis) shows a pattern of weak negative association between SYNTAX Score and Ca concentration, revealing any potential nonlinear aspects of the relationship. Patients with a higher Syntax score presented slightly lower Ca concentration.

### 3.3. Difference in Ca Level Between Patients with Stable CAD and Patients with ACS

Significant differences were observed between patients with ACS and stable CAD in LDL, TG, smoking status, Syntax score, PLR, and SII (Table 1). Patients with ACS presented significantly higher LDL, lower TG, and higher Syntax score, PLR, and SII; in this group, there were more active smokers.

Table 2 presents comparison between patients with stable CAD and detailed diagnosis of ACS. There were significant results (nonparametric Kruskal–Wallis test) for LDL and PLR (Dunn’s multiple comparisons test: patients with STEMI presented significantly higher values than patients with stable CAD, *p* = 0.016 and 0.029, respectively); TG (Dunn’s multiple comparisons test: patients with NSTEMI presented significantly lower values than patients with stable CAD, *p* = 0.025); and Syntax score (non-significant differences in Dunn’s multiple comparisons post hoc tests).

### 3.4. Correlation/Association Between Ca Level and Various Factors

**Figure 3:**
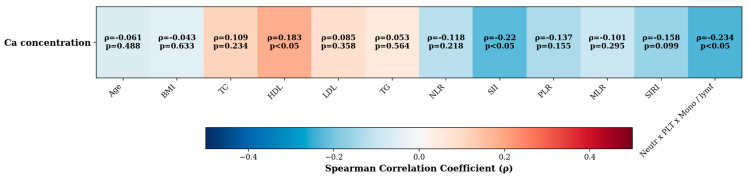
Spearman rank correlations (data did not meet the assumptions of normality) between Ca concentration (row) and 12 clinical/inflammatory variables (columns). Cell shading: blue → positive ρ, red → negative ρ (scale −0.5 to 0.5). Each box lists ρ and *p*-value.

Figure 3 presents the correlation between Ca level and various parameters. There was no significant correlation between Ca level and age, BMI, TC, LDL, TG, NLR, MLR, PLR, or SIRI (ρ between −0.16 and 0.11). Significant negative correlation was observed between Ca level and SII (*p* = 0.021) and between Ca level and neutrophils × PLT × monocytes/lymphocyte ratio (*p* = 0.014). A positive correlation was observed between Ca level and HDL (*p* = 0.046).

Women presented significantly higher Ca levels than men (Mann–Whitney test, *p* = 0.021). There were no significant differences in Ca level between patients with different smoking status (K-W: 1.545, *p* = 0.462), those with or without diagnosed hypertension (Mann–Whitney test, *p* = 0.095), hyperlipidemia (Mann–Whitney test, *p* = 0.789), or t2DM (K-W: 2.044, *p* = 0.359).

## 4. Discussion

The presented analysis of a group of patients with angiographically confirmed CAD showed no significant differences in the concentration of calcium in hair in the subgroups diagnosed with chronic and acute coronary syndrome. Furthermore, within the ACS subgroup, there was no significant correlation between hair calcium concentration and specific types of myocardial infarction, including those with and without ST-segment elevation or those with unstable angina. Numerous prospective studies involving hundreds of thousands of participants have shown a link between calcium concentration and the incidence of ACS. Specifically, these studies indicate that for every 0.1 mmol/l increase in calcium concentration, there is a corresponding 10% rise in the risk of experiencing a coronary event [8]. It should be noted, however, that these observations refer to the transient calcium concentration in serum, whereas the findings of our study are based on an analysis of long-term (6–8 weeks) calcium exposure, as reflected by its concentration in hair samples from patients with normal serum levels of this trace element. To date, only a few authors have attempted to assess the relationship between hair calcium content—or, more broadly, the long-term role of calcium—and the development of acute complications of coronary atherosclerosis, reporting inconsistent findings. A 1993 study indicated a correlation between the severity of coronary atherosclerosis and the concentration of trace elements in hair samples from autopsied individuals, highlighting a significant link with elements such as calcium [63].

It is important to emphasize that the aforementioned conclusions regarding occlusion, degree of stenosis, and disease progression, assessed within the three main coronary arteries, were associated with the calcium-to-potassium (Ca/K) ratio rather than calcium levels alone. Additionally, the study was conducted using post mortem material, which may have influenced the interpretation of the results. The connection between calcium and coronary atherosclerosis’s acute complications is poorly understood. Only a few studies have investigated this issue, and the findings have often been inconsistent [64,65]. Inconsistent data are similarly observed when estimating the risk of cardiovascular complications related to hair calcium levels [66,67,68]. Observations conducted by MacPherson et al. on patients from the UK suggest that calcium concentration in hair samples may be associated with the risk of CCS. This relationship appears to be largely influenced by environmental factors such as drinking water hardness and annual sunlight exposure. The highest hair calcium levels, hardest water, greatest sunlight exposure, and lowest CCS mortality were observed in individuals from south-east England—findings that stand in stark contrast to those reported in the Scottish population [67]. Nearly a decade later, Afridi et al. reported elevated calcium levels in biological samples, including hair, from patients with ACS compared to reference values; however, these differences did not reach statistical significance [69]. Considering both the results of our study and the findings of previously cited research, a definitive link between long-term calcium intake and myocardial infarction has not yet been established.

Moreover, our analysis revealed a negative correlation between calcium concentration in hair samples and the severity of coronary atherosclerosis. To date, this topic has been explored by only a few research groups. Similar conclusions indicating a negative correlation between the two variables were reported by Urbanowicz et al. It is worth noting that in contrast to our study, the authors excluded patients with three-vessel coronary artery disease and ACS from their analysis. Their findings, which suggest an association between lower calcium levels and more advanced atherosclerosis, apply only to cases of one- or two-vessel CCS [70]. Current research findings suggest a relationship between calcium concentration in hair and its content in bones, as estimated by bone mineral density (BMD). This association is particularly relevant to our study, considering the evidence indicating an intrinsic link between osteoporosis and atherosclerosis [71]. The analysis conducted by Soo-Jung Park et al. on a group of 55 Korean women over 20 years of age revealed significantly lower BMD and T-score in the L1–L4 vertebrae in the cohort with the highest calcium levels in hair [72]. Similar conclusions, indicating a correlation between hair calcium concentration and the severity of osteoporosis in a group of older women, were recently presented by Anatoly V. Skalny et al. [73]. The authors emphasize that this relationship is particularly pronounced in postmenopausal women. They discuss the connections between common causes and genes associated with osteoporosis and atherosclerosis, as well as the increased risk of cardiovascular disease mortality linked to BMD [74,75]. The results of the cited studies, which suggest an inverse correlation between hair calcium content and the degree of bone tissue mineralization, may support our observations indicating a negative association between calcium concentration and the severity of coronary atherosclerosis.

In conclusion, the relationship between calcium levels in hair samples, the progression of CCS, and the risk of ACS remains open to further investigation. Moreover, there is still insufficient evidence to support the use of this parameter as a marker of myocardial injury. Nevertheless, given the steady increase in the incidence of CCS, the role of proposed—particularly modifiable—atherosclerosis risk factors, including trace element supply, warrants further clarification.

In our study, the SII index was significantly higher in patients with ACS compared to those diagnosed with CCS [40,76,77]. The SII, based on platelet, neutrophil, and lymphocyte counts—cells with a well-established role in the pathogenesis of atherosclerosis—has been shown to assist in predicting major adverse cardiovascular events (MACEs) [78,79,80,81,82,83,84,85,86,87,88,89,90,91,92,93,94,95,96].

It is an important predictor of early thrombus formation in patients following ACS [97]. During both in-hospital and post-discharge follow-up, elevated SII was independently associated with MACEs and increased mortality [98]. Moreover, it proved to be the most reliable independent predictor of poor clinical outcomes in ACS patients, outperforming both the neutrophil-to-lymphocyte ratio (NLR) and the platelet-to-lymphocyte ratio (PLR) [49].

The observations presented herein indicate no significant correlation between the SIRI index and the diagnosis of ACS. This finding stands in contrast to both our previous results [99] and those reported by other researchers [100]. We believe that the lack of correlation between SIRI and ACS in our study is most likely due to the relatively small size of the analyzed cohort. In a 2022 study involving nearly 700 patients, a relationship between both SII and SIRI markers and ACS events was suggested, with significantly higher SIRI values observed in patients with STEMI [99]. Similarly, Hamrish Kumar Rajakumar et al. clearly identified SIRI as a predictive marker for ACS [100], while Wenjun Fan et al. considered it a predictor of increased risk of MACEs in patients undergoing PCI after myocardial infarction [101].

It should be emphasized that the role of monocytes, which are among the key factors influencing the value of the SIRI index, in the pathogenesis of atherosclerotic plaques [102,103], is also recognized by the scientific community. Recently, the significance of innate immune cells has been emphasized, as they have been demonstrated to retain a memory of inflammatory exposure, a phenomenon known as “trained immunity,” which contributes to the development of an inflammatory phenotype [104,105,106].

Importantly, in the context of atherosclerosis pathogenesis and the analyzed biomarkers, our findings demonstrate a negative correlation between calcium concentration in hair samples and the values of both SII and SIRI. To the best of our knowledge, this specific association has not yet been explored in previous studies. However, calcium signaling pathways have long been linked to the functioning of immune system cells, particularly in their activation, chemotaxis, and phagocytosis [107]. As demonstrated in the introduction, this bioelement plays a key role in the functioning of neutrophils [20,21], lymphocytes [22,23,24], monocytes [25,26,27], and platelets [28,29,30]—all of which are components of the low-grade inflammatory markers analyzed in this study.

Biomarkers based on leukocyte subpopulation, along with platelet counts, may serve as integrative tools for cardiovascular risk stratification, particularly in acute coronary syndromes. Given the documented role of calcium in regulating immune cell function involved in atherogenesis, and the inverse correlation between hair calcium levels and subclinical inflammatory markers, long-term modulation of calcium intake may contribute to the regulation of arterial wall inflammation.

Calcium acts as a context-dependent modulator in atherogenesis. As a key second messenger, it orchestrates endothelial and immune cell functions such as activation, adhesion, and apoptosis [108,109]. Under inflammatory conditions, elevated intracellular calcium promotes endothelial barrier disruption, leukocyte recruitment, and lesion progression [110]. In vascular smooth muscle cells (VSMCs), calcium-sensing receptor (CaSR) activation elevates cytosolic calcium, triggering NLRP3 inflammasome activation and contributing to vascular remodeling [111]. A similar mechanism in monocytes enhances systemic inflammation through calcium phosphate uptake [112].

Conversely, CaSR activation may also exert protective effects. Endothelial CaSR signaling downregulates VCAM-1, IL-6, and NLRP3 inflammasome activity, supporting endothelial stability and limiting inflammation [11].

Epidemiological evidence further suggests that calcium’s vascular effects depend on its source. Dietary calcium appears protective, whereas supplementation has been associated with increased coronary artery calcification (CAC) and vascular dysfunction [113].

Calcium functions as a bidirectional modulator in inflammation and atherogenesis. Its net effect—pro- or anti-inflammatory—is influenced by inflammatory status, cellular context, and receptor engagement. Understanding these calcium-dependent pathways may aid in identifying new therapeutic strategies in cardiovascular disease.

In the studied patient population, we observed a correlation between hair calcium content and the concentration of high-density lipoprotein (HDL) cholesterol, with no observed relationship with other lipid fractions. HDL, commonly referred to as “good cholesterol”, stimulates the transport of cholesterol from peripheral vessels to the liver, where it is subsequently excreted in the feces [114]. A relationship between calcium levels and the lipid profile was previously suggested by Vanaelst et al., who demonstrated a negative correlation between calcium and magnesium concentrations in hair samples and non-HDL cholesterol levels [115]. These observations may be explained by calcium-dependent signaling pathways, such as the inositol 1,4,5-trisphosphate (IP3)-Ca^2+^ pathway, the p38 mitogen-activated protein kinase (p38-MAPK) pathway, and the calmodulin-binding pathway, through which calcium promotes adipocyte differentiation and enhances energy expenditure [116].

The study has several significant limitations that should be considered when analyzing our results and attempting to validate them in future research projects. The cohort of patients enrolled in the study was characterized by a small sample size and limited demographic diversity, which diminishes the global applicability of the presented findings. We did not account for the influence of medications that modify calcium levels in the body, such as β-adrenolytics, angiotensin receptor blockers, angiotensin-converting enzyme inhibitors, and diuretics. Calcium content was analyzed only in serum and hair samples; expanding the observations to include analysis of calcium levels in urine could significantly enhance the value of the results. So far, specific factors determining the process of calcium deposition in the hair follicle have not been identified, which may influence the measurement of its concentration.

The lack of clearly defined calcium level ranges in hair samples within the general population has complicated the interpretation of the results. The assessment of inflammation was based solely on C-reactive protein (CRP) levels, without considering inflammatory cytokines and ferritin. The degree of atherosclerosis was assessed using the SYNTAX score, without analyzing the coronary artery calcium score, the type of statin used, or the duration of lipid-lowering treatment. Dietary habits and body composition analysis were also not taken into account.

In conclusion, no significant differences in calcium content in hair samples between patients with stable CAD and ACS were observed. However, a significant inverse correlation was found between calcium levels and the severity of coronary atherosclerosis. Additionally, patients with ACS presented with higher levels of subclinical inflammation, as assessed by the SII index. Considering the well-established role of calcium as a modulator of immune cell activity—demonstrated in numerous experimental and clinical studies—our findings may serve as a basis for further investigation into the potential link between long-term calcium status and inflammatory mechanisms involved in atherosclerosis. The observed inverse correlation between calcium levels and inflammatory markers (SII and SIRI) supports existing hypotheses suggesting that disturbances in calcium homeostasis may promote inflammatory responses and the progression of atherosclerotic lesions. Additionally, emerging data suggest a potential role of the heart–liver axis in modulating post-infarction inflammatory responses [117]. Acute myocardial injury may disrupt calcium homeostasis, which, in turn, can affect hepatocyte function and systemic immune activity.

Although the measurement of elemental content in hair is not currently used in routine cardiological diagnostics, it may hold potential as a complementary tool for assessing long-term mineral status. When combined with the evaluation of inflammatory biomarkers, this approach could contribute to a more comprehensive assessment of atherosclerosis risk and its clinical consequences.

Due to the observational nature of the study, the relatively small sample size, and the inability to establish causal relationships, these findings should be interpreted with caution. Further prospective studies involving larger cohorts and accounting for additional variables influencing calcium metabolism and inflammatory processes are warranted to verify and expand upon our observations.

## 5. Conclusions

In patients with normal serum calcium levels, no differences in hair calcium content were found between CCS and ACS groups or among ACS subtypes (STEMI, NSTEMI, UA). Lower hair calcium levels were associated with more advanced coronary atherosclerosis rated on the Syntax scale. Patients with ACS diagnosis exhibited significantly elevated SII values and nominally lower hair calcium concentration. In the overall cohort, hair calcium levels showed a significant inverse correlation with SII. These findings underscore the need for larger, well-designed studies to clarify the role of long-term calcium burden in low-grade inflammation and atherosclerosis pathogenesis.

## Figures and Tables

**Table 1:** Difference in selected parameters between patients with stable CAD and patients with ACS.

Variable	All Patients	Stable CAD (N = 65, 50%)	ACS (N = 65, 50%)	*p* *
Number (♀/♂)	36/94	20/45	16/49	0.433
Age (years)	65 (52–85)	63 (52–84)	66 (48–87)	0.333
BMI (kg/m^2^)	28 (23–37)	28 (23–38)	28 (22–35)	0.592
t2DM (yes/pre-diabetes/no)	41/7/82	23/3/39	18/4/43	0.623
TC (mg/dL)	165 (99–260)	159 (118–251)	168 (94–294)	0.160
HDL (mg/dL)	46 (29–68)	46 (32–67)	47 (29–68)	0.365
LDL (mg/dL)	89 (33–189)	77 (41–170)	101 (33–206)	0.009
TG (mg/dL)	110 (64–245)	122 (72–281)	99 (48–189)	0.006
Hyperlipidemia (yes/no)	53/67	28/33	25/34	0.697
Hypertension (yes/no)	112/18	55/10	57/8	0.612
Smoking (active/former/no)	38/17/75	17/15/33	21/2/42	0.003
Previous MI (yes/no)	38/52	22/43	16/49	0.247
Syntax score	14 (0–57)	9 (0–47)	15 (0–61)	0.011
NLR	2.3 (1.2–5.3)	2.1 (1.1–5.0)	2.4 (1.2–5.5)	0.232
MLR	0.35 (0.19–0.71)	0.34 (0.18–0.71)	0.36 (0.21–0.77)	0.756
PLR	105 (51–207)	95 (41–207)	119 (61–212)	0.003
SII	502 (186–1429)	434 (166–1210)	547 (195–1464)	0.028
SIRI	1.63 (0.74–5.55)	1.63 (0.75–5.77)	1.63 (0.72–5.55)	0.779
Neutro × PLT × Mono/Lym	350 (124–1644)	314 (124–1611)	404 (123–2032)	0.298
Ca (ppm)	321 (85–1290)	340 (70–1290)	315 (96–1121)	0.392

*—*p*-value for the comparison between patients with stable CAD and ACS: Mann–Whitney test used for continuous variables (data did not meet the assumptions of normality), Chi2 test used for categorical data; BMI—body mass index; t2DM—type 2 diabetes mellitus; TC—total cholesterol; HDL—high-density lipoprotein; LDL—low-density lipoprotein: TG—triglyceride; MI—myocardial infarction; NLR—neutrophil-to-lymphocyte ratio; MLR—monocyte-to-lymphocyte ratio; PLR—platelet-to-lymphocyte ratio; SII—Systemic Immune-Inflammation Index; SIRI—Systemic Inflammation Response Index; Neutro—Neutrophils; PLT—platelet; Mono—monocyte; Lym—lymphocyte; Ca—calcium.

**Table 2:** Difference in selected parameters between patients with stable CAD and patients with STEMI, NSTEMI, and UA.

Variable	Stable CAD	STEMI	NSTEMI	UA	*p* *
Number (♀/♂)	20/45	7/24	5/14	4/11	0.864
Age (years)	63 (52–84)	61 (45–89)	74 (56–84)	66 (59–87)	0.079
BMI (kg/m^2^)	28 (23–38)	28 (19–35)	27 (21–45)	29 (22–36)	0.496
t2DM (yes/pre-diabetes/no)	23/3/39	8/2/21	4/2/13	6/0/9	0.701
TC (mg/dL)	159 (118–251)	169 (135–283)	161 (70–245)	167 (94–311)	0.113
HDL (mg/dL)	46 (32–67)	47 (30–68)	43 (29–68)	46 (26–67)	0.439
LDL (mg/dL)	77 (41–170)	105 (63–188)	96 (27–175)	102 (33–228)	0.024
TG (mg/dL)	122 (72–281)	105 (68–205)	91 (43–157)	101 (43–189)	0.023
Hyperlipidemia (yes/no)	28/33	13/16	5/11	7/7	0.716
Hypertension (yes/no)	55/10	24/7	18/1	15/0	0.127
Smoking (active/former/no)	17/15/33	13/1/17	5/0/14	3/1/11	0.023
Previous MI (yes/no)	22/43	6/25	5/14	5/10	0.507
Syntax score	9 (0–47)	15 (2–52)	22 (0–69)	13 (0–36)	0.042
NLR	2.1 (1.1–5.0)	2.4 (1.2–8.1)	2.2 (1.1–3.1)	2.7 (1.8–3.9)	0.201
MLR	0.34 (0.18–0.71)	0.39 (0.21–0.91)	0.34 (0.21–0.56)	0.33 (0.21–0.55)	0.614
PLR	95 (41–207)	123 (61–223)	109 (61–190)	126 (51–160)	0.022
SII	434 (166–1210)	539 (241–2338)	522 (186–1464)	587 (337–865)	0.117
SIRI	1.63 (0.75–5.77)	1.66 (0.74–7.41)	1.46 (0.54–4.35)	1.52 (0.97–3.42)	0.871
Neutro × PLT × mono/lym	314 (124–1611)	427 (123–2032)	368 (95–2532)	352 (168–770)	0.646
Ca (ppm)	340 (70–1290)	322 (96–1793)	217 (71–581)	380 (165–1121)	0.225

*—*p*-value for nonparametric Kruskal–Wallis test (comparison between all presented groups of patients) used for continuous variables (data did not meet the assumptions of normality) or chi-squared test used for categorical data; BMI—body mass index; t2DM—type 2 diabetes mellitus; TC—total cholesterol; HDL—high-density lipoprotein; LDL—low-density lipoprotein: TG—triglyceride; MI—myocardial infarction; NLR—neutrophil-to-lymphocyte ratio; MLR—monocyte-to-lymphocyte ratio; PLR—platelet-to-lymphocyte ratio; SII—Systemic Immune-Inflammation Index; SIRI—Systemic Inflammation Response Index; Neutro—Neutrophils; PLT—platelet; Mono—monocyte; Lym—lymphocyte; Ca—calcium.

## Data Availability

Data can be provided by the corresponding author upon reasonable request.
